# Positioning of darunavir/cobicistat-containing antiretroviral regimens in real life: results from a large multicentre observational prospective cohort (SCOLTA)

**DOI:** 10.1186/s12981-019-0236-0

**Published:** 2019-08-26

**Authors:** Lucia Taramasso, Elena Ricci, Antonio Cascio, Laura Valsecchi, Barbara Menzaghi, Nicola Squillace, Paolo Maggi, Giuseppe Vittorio De Socio, Chiara Dentone, Giordano Madeddu, Giovanni F. Pellicanò, Leonardo Calza, Goffredo Angioni, Paolo Bonfanti, Antonio Di Biagio, CISAI Study Group, Paolo Bonfanti, Antonio Di Biagio, Elena Ricci, E. Sarchi, G. Chichino, C. Bellacosa, G. Angarano, L. Calza, B. Menzaghi, M. Farinazzo, G. Angioni, M. Gussio, B. M. Celesia, K. Falasca, A. Mastroianni, G. Guadagnino, F. Vichi, E. Salomoni, C. Martinelli, A. Di Biagio, L. Nicolini, G. Cenderello, P. Bonfanti, C. Molteni, G. F. Pellicanò, G. Nunnari, L. Valsecchi, L. Cordier, A. Parisini, G. Rizzardini, S. Rusconi, F. Conti, A. Bandera, L. Taramasso, A. Gori, D. Motta, M. Puoti, N. Squillace, G. M. Migliorino, P. Maggi, S. Martini, A. Cascio, M. Trizzino, R. Gulminetti, G. V. De Socio, D. Cibelli, G. Parruti, C. Dentone, G. Madeddu, M. S. Mameli, G. Orofino, M. Guastavigna

**Affiliations:** 1 Department of Health Science (DISSAL), Infectious Disease Clinic, University of Genova, Genoa, Italy; 2 Infectious Diseases Unit, Department of Internal Medicine, Fondazione IRCCS Ca’ Granda Ospedale Maggiore Policlinico, Via Francesco Sforza 35, 20122 Milan, Italy; 3 Dipartimento Donna-Bambino-Neonato, Fondazione IRCCS Ca’ Granda Ospedale Maggiore Policlinico, Milan, Italy; 4 Department of Health Promotion Sciences, Maternal and Infant Care, Internal Medicine and Medical Specialties (PROMISE) - Infectious Disease Unit, Policlinico “P. Giaccone”, University of Palermo, Palermo, Italy; 5 Infectious Disease Unit (I Divisione), ASST Fatebenefratelli Sacco, Milan, Italy; 6 Infectious Disease Unit, ASST della Valle Olona, Busto Arsizio, Italy; 7 Infectious Disease Unit, San Gerardo Hospital, University of Milano-Bicocca, Monza, Italy; 8 Infectious Diseases Clinic University of Campania “Luigi Vanvitelli”, Neaples, Italy; 9 Infectious Disease Unit, Santa Maria Hospital, Perugia, Italy; 10 Infectious Disease Unit, Sanremo Hospital, Sanremo, Italy; 11 Unit of Infectious Diseases, Department of Medical, Surgical and Experimental Sciences, University of Sassari, Sassari, Italy; 12 Unit of Infectious Diseases, Department of Human Pathology of the Adult and the Developmental Age “G. Barresi”, University of Messina, Messina, Italy; 13 Department of Medical and Surgical Sciences, Unit of Infectious Diseases, ‘Alma Mater Studiorum’ University of Bologna, S. Orsola-Malpighi Hospital, Bologna, Italy; 14 Infectious Disease Unit, SS Trinità Hospital, Cagliari, Italy; 15 Infectious Disease Unit, Ospedale A. Manzoni, Lecco, Italy; 16 Infectious Diseases Clinic, Policlinico Hospital San Martino, Genoa, Italy

**Keywords:** Darunavir/cobicistat, Dual, Durability, Tolerability, CISAI, Adverse events

## Abstract

**Background:**

Study aim was to evaluate the safety and durability of darunavir/cobicistat (DRV/c) in a real life setting.

**Methods:**

Multicentre prospective cohort study performed in the context of SCOLTA (Surveillance Cohort Long-Term Toxicity Antiretrovirals). Patients were evaluated at baseline, week 24 and 48. Changes were evaluated using the paired *t* test or signed rank test. The multivariable analysis was performed using a general linear model, after ranking of not normally distributed variables.

**Results:**

A total of 249 patients were included, 72 (29%) were in DRV/c-based dual therapies (DT). Hypercholesterolemia, HC, (total cholesterol (TC) ≥ 200 mg/dL or low density-C (LDL-C) ≥ 130 or statin use) was present in 121 (48.6%) and hypertriglyceridemia, (triglycerides (TG) ≥ 200 mg/dl or fibrate use) in 41 (16.5%) patients. Blood lipid profile did not change significantly in either the global population or patients with HC. After a median observation of 17 months (IQR 13–20), 59 (25.3%) patients discontinued DRV/c, of which 13 were in DT. The durability DT resulted higher than that of triple therapy (log-rank test p = 0.01). Main reasons for stopping DRV/c were simplification (15 patients), adverse events (13 patients), planned discontinuation for treatment initiation with DAA (4 patients), treatment failure (2 patients); death (2 patients), other causes (10 patients). Twenty-six were lost to follow-up.

**Conclusions:**

DRV/c was safe and well tolerated. Dual therapies showed a better profile of tolerability and a longer durability compared to triple therapies.

## Background

The first arrival of protease inhibitors (PI) in 1996 [1] has been one of the turning points in the history of AIDS. For years, PI have been the preferred third agents for combined antiretroviral therapies (cART) [2] but, in the more recent period, only darunavir (DRV) (boosted with either ritonavir or cobicistat) is the preferred PI in first-line cART, in combination with two nucleos(t)ide reverse transcriptase inhibitors (NRTI) in current EACS guidelines [3], and similarly, DRV is the preferred PI in certain clinical situations in DHHS and Italian guidelines. [4, 5]. The PIs remain instead the cornerstone of salvage therapies, in non-adherent patients, both naïve or experienced [6, 7]. The novel co-formulation of darunavir/cobicistat (DRV/c) constitutes a new step forward towards improving convenience and adherence without loss of virological efficacy. Moreover, DRV/c co-formulation offers the possibility of reducing the side effects classically linked to ritonavir boosting use, including multiple drug interactions, metabolic and gastrointestinal effects [8, 9]. Clinical trials of DRV/c showed promising results of efficacy and tolerability in both first line [10, 11] and switch strategies [12, 13], with high viral response also in experienced patients with history of previous virological failures and exposure to multiple antiretroviral classes [10].

Moreover, triple combinations of antiretrovirals have been the standard treatment for HIV infection, but the recent advent of more potent and safer antiretrovirals has renewed the interest for simpler HIV regimens. However, besides clinical trials, data on real-life experience with DRV/c both in dual or standard triple therapy are still scarce, and mainly based on retrospective studies [14, 15] or restricted to mono or dual therapies [16].

The aim of the present study is to evaluate DRV/c durability and tolerability in dual as well as in triple therapy, using data from the SCOLTA prospective study cohort, a real-life setting.

## Materials and methods

The SCOLTA (Surveillance Cohort Long-Term Toxicity Antiretrovirals) project is a multicentre and observational study, started in 2002, that follows HIV-infected people who start a new drug prospectively, with the aim of identifying toxicities and adverse events in real-life setting.

For the present study, all patients aged > 18 years who started DRV/c in the participating centers, in either first line or switch strategies, were considered eligible.

Demographic, clinical and laboratory data, including CD4+ T-cell count (CD4), HIV-RNA, creatinine level and metabolic data such as total cholesterol (TC), triglycerides (TG), LDL-cholesterol (LDL), HDL-cholesterol (HDL), were prospectively collected in anonymous form in a central database at the moment of initiation of DRV/c and every 24 weeks. For patients experienced to antiretroviral treatment, data on the previous antiretrovirals and cumulative exposure to antiretroviral therapy were collected.

Estimated glomerular filtration rate (eGFR) was calculated using Cockroft-Gault equation [17]. Hypercholesterolemia (HC) was defined as TC ≥ 200 mg/dL or LDL ≥ 130 or statin use. Hypertriglyceridemia (HT) was defined as TG ≥ 200 mg/dl or fibrate use.

Patients were described using frequency for categorical variables and mean (standard deviation, SD) or median (interquartile range, IQR) for continuous variables. DRV-containing dual and triple therapy groups were compared using heterogeneity Chi square test (of Fisher’s or Mantel-Hanszel test as appropriate), analysis of variance or Mann-Withney U test, respectively, as regards baseline characteristics. Patients were evaluated at baseline (T0), week 24 (T1) and week 48 (T2). Change from T0 was evaluated using the paired t-test for continuous variables if normally distributed and the signed rank test if not. Changes in eGFR, CD4, and lipids at T1 and T2 were subsequently compared between patients on dual and on triple therapy using analysis of variance or Mann-Withney U test, respectively.

Changes in eGFR were also compared in groups of patients taking tenofovir disoproxil fumarate (TDF) or not as part of their ART regimen, using the analysis of variance to perform the between-groups comparison.

The multivariable analysis was performed using a general linear model, after ranking of not normally distributed variables, if any. The durability of DRV/c treatment was defined as the time on continuous DRV/c use. The discontinuation or changing of other components of the ART regimen was not considered in durability analysis. Hazard ratio for discontinuation of DRV/c were calculated from fitting a Cox regression model and durability was represented throughout Kaplan–Meier plot. Differences were considered significant for p values < 0.05.

Grade 3–4 adverse events and treatment interruptions were recorded and detailed in the central database when they occurred [18]. Data collection started in May 2016, and continued till present (last data merger in March 2019).

The study protocol was approved by the local ethics committees of the participating centres and written consent was obtained from all participants.

## Results

We identified 249 HIV-infected patients who started DRV/c containing antiretroviral regimens. The general characteristics of the patients are showed in Table 1.

**Table 1:** Baseline characteristics of 249 patients enrolled in the DRV/COBI cohort

	Dual therapy		Triple therapy		P
	N	%	N	%	
Enrolled patients	72	28.9	177	71.1	
Female	28	38.9	39	22.0	0.007
Risk factor for HIV acquisition					
Sexual	30	41.7	103	58.2	
Intravenous drug use	29	40.3	46	26.0	
Transfusion/vertical/unknown	13	5.6	28	1.1	0.04
Ethnicity					
Caucasian	70	97.2	165	93.2	
Other	2	2.8	12	6.8	0.21
Naive	1	1.4	8	4.5	0.45
CDC stage					
A	15	20.8	47	26.6	
B	40	55.6	68	38.4	
C	17	23.6	62	35.0	0.59*
HIVRNA > 50 copies/mL at T0**	11	15.5	12	7.1	0.04
Previous regimen including**					
PI	61	85.9	161	95.3	0.01
NNRTI	15	21.1	4	2.4	< 0.0001
Hypercholesterolemia	44	61.1	77	43.5	0.03
Hypertriglyceridemia	11	15.3	30	16.9	0.67

	Mean or median	SD or IQR	Mean or median	SD or IQR	
Mean age, SD (years)	50.2	10.8	48.2	10.1	0.17
Mean CD4, SD (cells/mm^3^)	625	381	581	343	0.37
Median time on PI, IQR (years)	8.8	5.9–11.5	5.8	2.5–10.0	0.001
Median time on NNRTI, IQR (years)	2.4	0.9–4.8	1.6	0.1–5.3	0.12
Median time on ART, IQR (years)	18.1	7.8–21.0	9.0	3.2–17.9	< 0.0001
Mean eGFR, SD (mL/min)	94.2	20.3	96.3	23.6	0.50

SD: Standard deviation; PI: protease inhibitors; NNRTI: non nucleoside reverse transcriptase inhibitors; IQR: interquartile range; eGFR: estimated glomerular filtration rate

* Mantel–Haenszel Chi square test

** Experienced patients, n = 240

Among them, 182 were males (73.1%), 235 Caucasian (94.4%). Mean age was 48.0 (± 10.4) years, similar in both sexes. Nine patients (3.6%) were cART naïve.

**Fig. 1:**
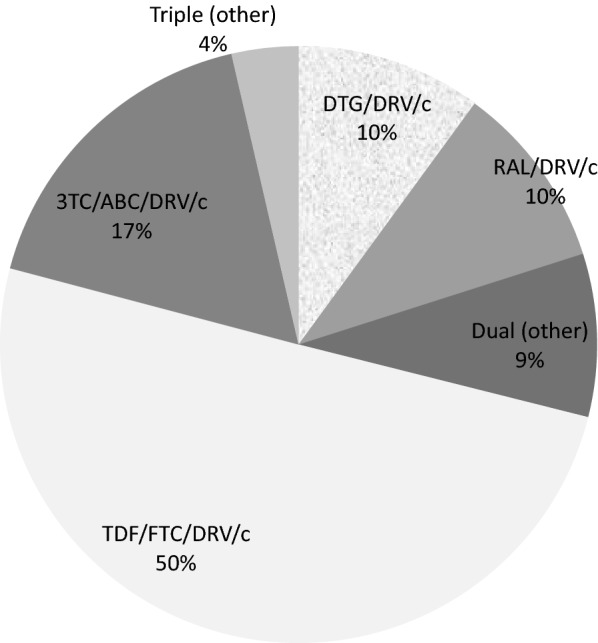
Antiretroviral therapy drug regimens in patients initiating darunavir/cobicistat (DRV/c). 3TC: lamivudine; ABC: abacavir; DRV/c: darunavir/cobicistat; DTG: dolutegravir; FTC: emtricitabine; RAL: raltegravir; TDF: tenofovir

Seventy-two (28.9%) were in DRV/c-based dual therapies and 177 (71.1%) in DRV/c triple therapy (Fig. 1); 76.4% and 77.4% respectively switched to DRV/c from regimens already including DRV.

Among them (N = 192 subjects) the median time on previous DRV treatment was 26 months (IQR 14–44). Follow up at 24 and 48 weeks was available for 239 (96.9%) and 181 (72.7%) patients, respectively. Sex and risk factor for HIV infection were different by groups, as well as time on previous PI and ART in general. Patients on dual therapy were also more likely hypercholesterolemic. On the whole, HC was present in 48.6% and HT in 16.5% of patients, similar in dual and triple therapy groups. At 24 and 48 weeks after DRV/c initiation CD4 increased, without significant differences between dual and triple therapy (Table 2).

**Table 2:** Changes in estimated glomerular filtration rate (eGFR), CD4+ T cell count (CD4), and lipids at 24 and 48 weeks after starting darunavir/cobicistat

	Dual therapy		Triple therapy		P between groups
	Mean	SD	Mean	SD	
eGFR mL/min					
T0	94.2	20.3	96.3	23.6	
T1	90.2	19.0	88.7	21.7	
T2	86.7	17.7	87.7	18.6	
T1-T0 (mean, SE)	− 4.6*	1.8	− 7.6*	1.3	0.19
T2-T0 (mean, SE)	− 7.6*	1.9	− 8.5*	1.4	0.71
CD4 cells/mm^3^					
T0	625.2	381.0	580.6	342.9	
T1	653.3	309.2	641.0	364.4	
T2	712.6	326.0	664.1	365.0	
T1-T0 (mean, SE)	18.5	30.5	60.8*	15.6	0.22
T2-T0 (mean, SE)	35.3	26.7	64.2*	19.1	0.38
HDL mg/dL					
T0	51.0	16.6	47.0	14.8	
T1	50.2	15.6	47.9	14.8	
T2	52.8	16.3	47.3	15.2	
T1-T0 (mean, SE)	− 1.2	1.0	1.2	0.8	0.07
T2-T0 (mean, SE)	1.8	2.2	0.7	1.2	0.62
TC mg/dL					
T0	200.0	47.6	187.3	42.5	
T1	199.4	45.8	190.0	40.8	
T2	204.5	45.3	192.5	46.6	
T1-T0 (mean, SE)	− 1.2	3.6	3.6	2.3	0.24
T2-T0 (mean, SE)	0.1	5.0	2.4	3.3	0.69
TG mg/dL					
T0 (median, IQR)	128	92–169	124	88–168	
T1 (median, IQR)	133	97–177	111	86–167	
T2 (median, IQR)	145	90–183	120	86–194	
T1-T0 (mean, SE)	32.3	31.7	− 5.7	4.4	0.24
T2-T0 (mean, SE)	4.3	8.6	3.3	6.5	0.92
TC/HDL					
T0	4.20	1.34	4.27	1.37	
T1	4.26	1.39	4.25	1.38	
T2	4.11	1.21	4.31	1.29	
T1-T0 (mean, SE)	0.07	0.08	− 0.05	0.08	0.31
T2-T0 (mean, SE)	− 0.13	0.14	− 0.05	0.09	0.62

SD: Standard deviation; SE: standard error; T0: baseline; T1: 24 week follow up; T2: 48 week follow up; TC: total cholesterol, TG: triglycerides; HDL: HDL cholesterol

* P for change from baseline < 0.05

As compared to baseline, eGFR significantly decreased at T1 and T2. Comparing eGFR trend in groups of patients on tenofovir (122, 49.2%) as potentially kidney-damaging drug we found that eGFR change from baseline was more marked in patients taking tenofovir than in others: − 9.5 ± 1.6 ml/min (p < 0.0001), and − 4.2 ± 1.4 ml/min (p = 0.003), significant in the between-groups comparison (p = 0.01). At T2, this difference would narrow, and eGFR was − 9.1 ± 1.8 and − 7.5 ± 1.5 ml/min respectively, similar between groups (p = 0.50).

Blood lipid profile did not change significantly in either the global population or patients with HC. No difference was found in TC, HDL, TC/HDL and TG change between patients in dual or in triple therapy (Table 2). Adjusting for factors that were different at baseline did not change our findings.

After a median observation of 17 months (IQR 13–20), 72 (28.9%) patients discontinued DRV/c, of which 16 (22.2%) were on dual and 56 (31.6%) on triple therapy.

**Fig. 2:**
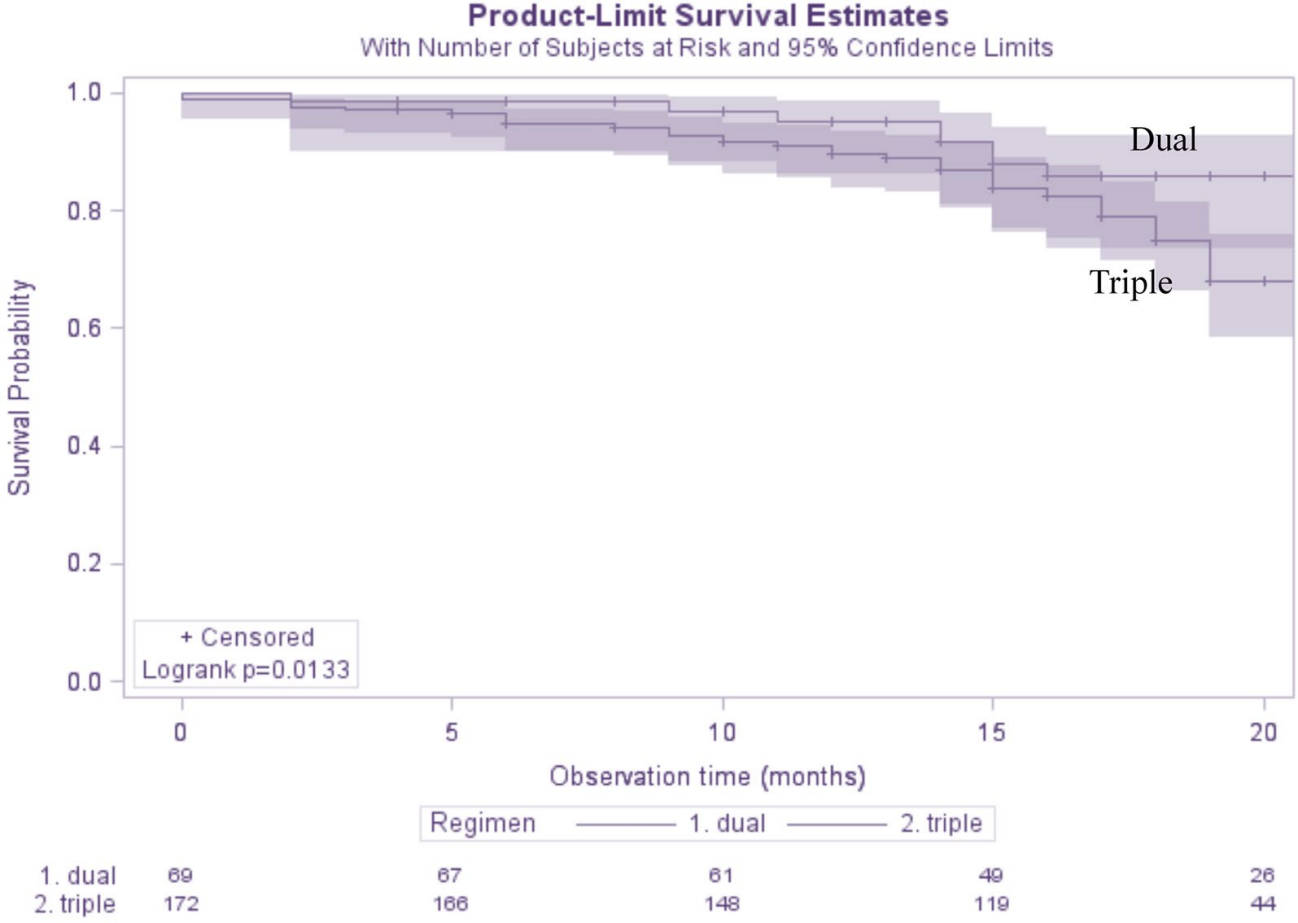
Durability of dual and triple combined antiretroviral regimens containing darunavir/cobicistat (DRV/c). Patients were censored when stopping DRV/c

The durability of dual regimens resulted higher than that of triple therapies (log-rank test p = 0.01, Fig. 2).

However, after adjusting for age, sex, risk factor for HIV acquisition, CDC stage at initiation and years on ART, the protective role of dual therapy was no longer significant (hazard ratio for interruption 0.70, 95% confidence interval 0.39–1.27, triple regimen as the reference).

Discontinuations due to adverse events were 13 (2 renal, 3 gastro-intestinal, 1 allergy, 1 asthenia, 3 dyslipidemia, 1 osteoporosis, 1 central nervous system, 1 undefined), 15 discontinued for simplifications or pro-active switches. Two patients discontinued DRV/c for treatment failure; 2 died (1 lung cancer, 1 unknown reason). Four interrupted because of drug–drug interactions, 8 for low adherence/patient’s preference. Twenty-six were lost to follow-up. Most adverse events and simplifications occurred in the triple regimen group (Table 3).

**Table 3:** Reasons for interruption of darunavir/cobicistat

	Dual therapy		Triple therapy		Total	
	N = 72	%	N = 177	%	N = 249	%
Adverse events	1	1.4	12	6.8	13	5.2
Simplification/proactive switch	1	1.4	14	7.9	15	6.0
Patient’s preference/low adherence	2	2.8	6	3.4	8	3.2
Therapeutic failure	2	2.8	0	0.0	2	0.8
Death	0	0.0	2	1.1	2	0.8
Drug-drug interaction	1	1.4	3	1.7	4	1.6
Other reasons	1	1.4	1	0.6	2	0.8
Lost to follow-up	8	11.1	18	10.2	26	10.4

## Discussion

This study investigated the safety and durability profile of DRV/c in a large prospective cohort (SCOLTA). Our results showed DRV/c was well tolerated and adverse events leading to discontinuations occurred in 13 patients, namely 5% of the study population. In the group of patients on triple therapy, simplification was the principal cause of discontinuation (14 patients on triple therapy vs. 1 patient on dual therapy). Of note, DRV/c has demonstrated low rate of virological failure (2 patients, 2.8%).

The rate of discontinuations due to adverse events was higher than that seen in EMERALD (i.e. 1%) and AMBER (i.e. 2%) trials [10, 12], but similar to real-life studies on DRV/ritonavir (i.e. 3–5%) [11, 19], suggesting, possibly, a lower threshold of tolerance for adverse events, or also a more difficult-to-treat population, in the context of real-life studies compared to clinical trials. Regarding the metabolic aspects of DRV safety, PI and, in particular, ritonavir-boosted PI, have been associated in the past to altered insulin sensitivity and unfavourable metabolic profile [20]. However, in a previous study, the switch from ritonavir to cobicistat was followed by a significant reduction in TC, LDL, but also of HDL, and TG levels in patients with HC [14]. In our prospective observation, despite most patients were switching from ritonavir boosted PI to DRV/c, we did not observe significant change in lipids, even in the sub-analysis focused on patients with higher baseline triglycerides and cholesterol levels, which were those expected to experience the greatest lipid changes [14, 21]. These results were consistent with previous studies including a large proportion of patients on ritonavir-boosted PI regimens who switched to DRV/c [12, 16], although lipid changes could have been partially confounded by the presence or removal of tenofovir disoproxil fumarate (TDF) from the regimen after the switch, with consequent lost of its lipid lowering effect [22]. Another issue related to the use of cobicistat is its inhibition of the tubular secretion of creatinine, owing to an expected apparent decrease of average 10 mL/min in the eGFR [23]. In the SCOLTA cohort, a reduction of eGFR was found, more evident after 6 months in people taking tenofovir disoproxil fumarate (TDF). A worse eGFR trend in course of TDF associated to cobicistat was also noticed in other studies [10, 12], suggesting the possibility of a TDF-boosting effect of cobicistat, also previously hypothesized for ritonavir [24, 25]. Indeed, DRV/c is expected to increase TDF plasma concentration due to P-glycoprotein inhibition, although there are currently inadequate data to determine whether TDF and DRV/c co-administration could be associated with highest risk of renal toxicity [26]. However, the eGFR decline at T2 was similar in patients who were tacking TDF or not.

Finally, two out of 249 patients (< 1%), experienced virological failure in the observation period (median 17 months), similar or even lower when compared with previous data [10, 12, 19].

Despite this, the total discontinuation rate was unexpectedly high, with 29% of the study population who stopped DRV/c, often for reasons other than adverse events, namely treatment simplifications, proactive switches or drug interactions. Those data could potentially be different with the arrival of the new single tablet formulation of DRV/c and tenofovir alafenamide (TAF), that could bring to lower rate of simplification thanks to the advantage of a single-pill regimen and the excellent safety profile of its TAF component [10, 12, 27]. On the other hand, the ageing of the HIV infected population and the desire of minimizing the possible metabolic effects of ART could be another important reason for proactive PI withdrawal from the regimen [28].

Of note, dual therapies showed a lower number of discontinuations due to any cause and also to adverse events. A possible explanation could be that the current direction of the modern HIV care is an optimization and a customization of the treatment, with dual therapies based on PI being tailored on patients needs. [29] On the contrary, PI triple therapy might be more generally prescribed to anyone, but demonstrated lower durability [30, 31], mainly for simplification or tolerability issues. Indeed, PIs have now many competitors in the modern ART scenario, including integrase inhibitors and rilpivirine, characterized by good tolerability and high durability [32–35]. In fact, despite DRV/c can be used in both naïve and experienced patients, our results showed as, in real life, it was mainly used in subjects switching from antiretroviral treatments already containing PI.

The present study has some limitations. First, a large number of heterogeneous ART regimens were included, comporting a possible confounding role of the accompanying backbones, if any. Second, SCOLTA protocol does not collect genotypic resistance data, and thus a further characterization of the study population and, in particular, of patients taking dual regimens, was not possible. Third, 48 weeks follow up was not available for a part of patients, limiting the power of some results.

## Conclusions

To our knowledge, this study is one of the largest prospective studies available to date on DRV/c use in a real-life context. The study included a large number of patients with a long history of past antiretroviral therapy, allowing the prospective evaluation of DRV/c safety and efficacy in a complex study population, likely similar to the one in care, and in treatment with PI, in clinical settings. DRV/c was safe and well tolerated in SCOLTA cohort and only 2 patients discontinued it due to virological failure. Dual therapies showed a better profile of tolerability and a longer durability compared to triple therapies.

## Data Availability

The dataset used and analyzed during the current study is available from the corresponding author on reasonable request.
